# Group Formation and the Evolution of Human Social Organization

**DOI:** 10.1177/17456916231179156

**Published:** 2023-07-14

**Authors:** Carsten K. W. De Dreu, Jörg Gross, Angelo Romano

**Affiliations:** 1 Department of Psychology, Leiden University; 2 Department of Psychology, University of Zurich

**Keywords:** social evolution, public goods, group processes, intergroup relations

## Abstract

Humans operate in groups that are oftentimes nested in multilayered collectives such as work units within departments and companies, neighborhoods within cities, and regions within nation states. With psychological science mostly focusing on proximate reasons for individuals to join existing groups and how existing groups function, we still poorly understand why groups form ex nihilo, how groups evolve into complex multilayered social structures, and what explains fission–fusion dynamics. Here we address group formation and the evolution of social organization at both the proximate and ultimate level of analysis. Building on models of fitness interdependence and cooperation, we propose that socioecologies can create positive interdependencies among strangers and pave the way for the formation of stable coalitions and groups through reciprocity and reputation-based partner selection. Such groups are marked by in-group bounded, parochial cooperation together with an array of social institutions for managing the commons, allowing groups to scale in size and complexity while avoiding the breakdown of cooperation. Our analysis reveals how distinct group cultures can endogenously emerge from reciprocal cooperation, shows that social identification and group commitment are likely consequences rather than causes of group cooperation, and explains when intergroup relations gravitate toward peaceful coexistence, integration, or conflict.

As for many other social species, group living provides *Homo sapiens* with levels of safety and prosperity that individuals can hardly achieve in isolation (Ostrom, 1998). Groups may contain as few as three individuals or as many as hundreds, can exist for a few hours or bind its members for most of their lifetime, and can be simple or exceedingly complex in their social organization. Regardless of their form and raison d’etre, individuals benefit from well-functioning groups and can be hurt—both mentally and physically—when their groups function poorly and disintegrate. Accordingly, psychological science has extensively addressed (a) what motivates individuals to join existing groups and prevents them from being excluded (e.g., Baumeister & Leary, 1995; Williams, 2000), (b) what allows existing groups to work and perform (De Dreu et al., 2008; Faber et al., 2017; Ilgen et al., 2005), and (c) what makes group members cooperate and resist the temptation to free ride on the public goods provided by others (Van Dijk & De Dreu, 2021; Van Lange et al., 2013).

What remains largely unaddressed in psychological science is how groups emerge and self-organize their internal dynamics and external relations: How do groups form ex nihilo, and how do groups evolve from simple to sometimes complex and multilayered collectives, such as fraternities within student societies, work units within companies, and neighborhoods within cities? Here we fill this gap and trace the evolution of human social organization to a succinct set of psychologically plausible behavioral mechanisms. Doing so scaffolds theory and research on existing groups and collectives, sheds new light on the origins and functions of well-documented phenomena such as homophily and group identification, and reveals parochial prosociality as a cause of group disintegration and intergroup conflict.

We proceed as follows. First, we invoke (fitness) interdependence theory to understand when and why strangers initiate costly helping and how (in)direct reciprocity and reputation-based partner selection leads them to form social ties and stable groups that create and maintain beneficial “club goods” (Figs. 1a and 1b). Second, we synthesize the literature on how collective action problems give rise to (in)formal social institutions such as leadership, socialization practices, shared norms, and role specializations (Fig. 1c). Third, we examine how reciprocity and reputation-based partner selection facilitates the merging of groups into larger, multilayered collectives (Fig. 1d) or, alternatively, remain parochial with as likely consequences “us-versus-them” thinking and intergroup conflict (Fig. 1e). We conclude by summarizing our findings and framework.

**Fig. 1.**
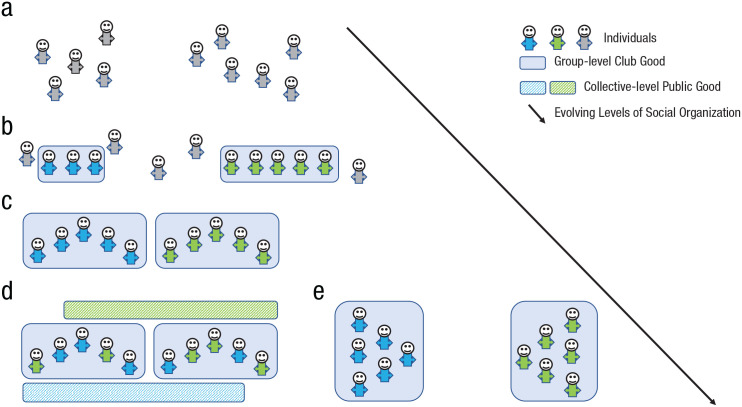
Human social organization from unconnected strangers (as an imaginary “state of nature”; a) that form groups to create and sustain local club goods (b) with social institutions and leadership structures (c). Groups can either merge with (d) or compete against (e) neighboring groups.

## The Formation and Evolution of Groups

Imagine a population of unrelated individuals that, when given a choice, prefer actions that maximize their own payoff (e.g., wealth, reproductive success). Imagine further that those individuals are randomly paired and can decide to provide benefit *b* to their partner at a personal cost *c*, with *b* > *c* > 0. In such a situation, helping will not be observed because incurring *c* reduces the helper’s payoff compared with not helping. And without helping, individuals remain independent, unbound to each other by past or future. In populations of strict payoff-maximizing individuals, how would social ties form and groups emerge?

### From individuals to groups: social interdependence and altruistic helping

Humans deviate from the imaginary payoff-maximizers in the above example—they help others at sometimes substantial cost to themselves, even when others are complete strangers and cannot return the favor. For example, when given the choice to donate money to an anonymous other, individuals on average give 28% of their resources, and 63% of all individuals give at least something (Engel, 2011). Typically, this altruistic helping observed in dictator games is explained by assuming that humans care about others’ payoff—they hold social preferences (Fehr & Schmidt, 1999; Hare, 2017; Van Lange, 1999). However, this explanation requires a compelling theory of why social preferences evolve in the first place, because any unconditional act of helping is exploitable and strictly dominated by selfishness from a payoff-maximizing perspective. Social preferences provide a proximate explanation for altruistic helping but ultimately cannot explain why strangers engage in altruism and cooperation (Geoffroy & André, 2021).

An ultimate mechanism for altruistic helping is suggested in fitness-interdependence theory (Aktipis et al., 2018; Balliet et al., 2017; Roberts, 2005). Fitness interdependence refers to the degree to which two individuals affect each other’s future payoffs. One factor that increases fitness interdependence is genetic relatedness, because facilitating the survival of kin helps with the transmission of one’s own genes to the next generations. Inclusive-fitness theory (Hamilton, 1964; see also Bourke, 2014; Croft et al., 2021), for example, proposes that individuals condition their helping on the genetic relatedness *r* between the individual actor and their partner (with 0 ≤ *r* ≤ 1). Whereas pure strangers with *r* = 0 should not help, individuals help with nonzero probability those with whom there is genetic relatedness, for example, because they are siblings (*r* = 0.5). The argument is that with genetic relatedness, other’s fitness indirectly benefits the helping individual, and this may partially or wholly offset the cost of helping (Smith, 1964). Fitness-interdependence theory expands this reasoning to any sort of situations in which the fitness of one individual—or payoffs more generally—can affect the fitness of another individual. Hence, fitness-interdependency theory includes, but is not limited to, genetic relatedness (Cronk et al., 2019).

**Fig. 2.**
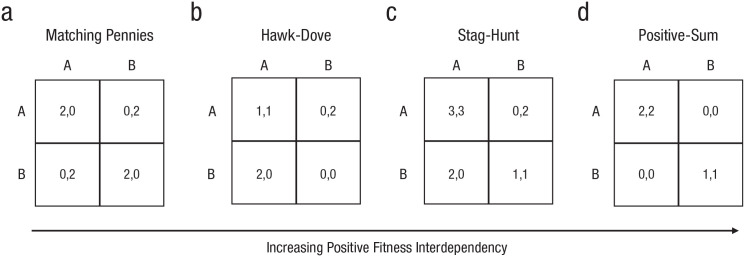
Fitness interdependency captured in exemplary games of strategy. Individual payoffs (left: row player; right: column player) depend on the combination of one’s own and another’s action (A or B). Across games ordered from matching pennies (a) to positive sum (d), there is an increase in positive correlation between the individual payoffs of the row and column player (specifically, an increase in the payoff by the column player corresponds to an increase in the payoff by the row player and vice versa).

Fitness interdependence can be captured with games of strategy (Van Dijk & De Dreu, 2021). In its simplest form, a game involves two individuals, each with two possible actions to choose from. In some games, individual payoffs are negatively correlated with those of the interaction partner—an increase in the fitness of one individual tends to be associated with a decrease in the fitness of the other (Roberts, 2005). Prominent examples include the matching-pennies and hawk-dove (also called “chicken-dilemma”) games (Figs. 2a and 2b). When there is negative fitness interdependence, helping can reduce own payoffs and/or can be exploited by the partner. This is different in games in which an increase in the fitness of one individual (can) coincide with an increase in the fitness of the other individual. Examples include stag-hunt (or “assurance”) and pure coordination games (Figs. 2c and 2d). When there is positive fitness interdependence, the action that serves one individual best is also preferred by the other and can be initiated without the risk of exploitation (Cronk et al., 2019; Leigh, 2010; Taborsky et al., 2016; see also Deutsch, 1973; Kelley & Thibaut, 1978).

Fitness interdependency, and the “game” strangers play, depend partly on the natural environment (Aktipis et al., 2018; Jaeggi et al., 2016). Resource scarcity, for example, can induce negative fitness interdependence and promote competition. Conversely, shared environmental threats, subsistence style, and opportunities for mutual gain can create positive interdependence and promote cooperation (Alvard & Nolin, 2002; Deutsch, 1973; Kelley & Thibaut, 1978). At the proximate level, sociocultural factors can likewise influence (assumptions about) the degree to which, and how, one’s own and another’s fitness are correlated (Columbus et al., 2021; Halevy et al., 2012). For example, people may (assume they) have more positive interdependence when they share a common fate (Ayers et al., 2023; Tjosvold, 1998), are physically close rather than distant (Carsten, 1995; Columbus et al., 2021), and are phenotypically similar rather than dissimilar (Hammond & Axelrod, 2006a; Mateo & Johnston, 2000; Platek et al., 2004). Fitting these possibilities, altruistic helping and cooperation increase when people share a common fate (Lojowska et al., 2023), are physically proximate (Buchan et al., 2002; Handley & Mathew, 2020), or are more rather than less similar (Balliet et al., 2014; Bian & Baillargeon, 2022; Lane, 2016).

**Fig. 3.**
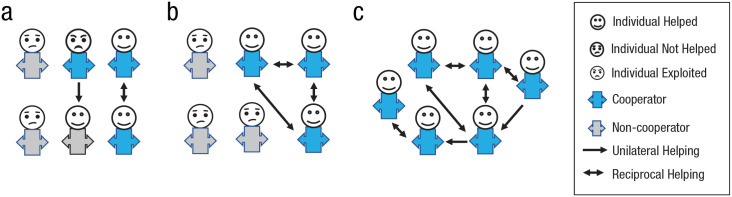
Cooperation and group formation. Strangers helping each other (blue bodies; a) more likely cooperate again, and others may seek partners with positive reputations for cooperation (b). This can escalate and spread cooperation and create groups as containers of bounded generalized reciprocity (c).

Strangers helping each other create mutual gain (i.e., 2*b* > 2*c*; Fig. 3a). This has two downstream consequences. First, direct reciprocity creates and strengthens social ties among strangers (Box 1; see also Jaeggi & Gurven, 2013). This can lead individuals to seek out further interactions—reciprocity can move individuals from a one-shot game into repeated interactions in which they share a past and possible future. In repeated interactions, cooperation can stabilize even when it can be exploited, especially when the probability of future interactions is high (Bó, 2005; Trivers, 1971; Van Veelen et al., 2012). The future cost of losing an interaction partner (either because the partner also starts to defect or is looking for other interaction partners) can outweigh the benefits of exploitation (Delton et al., 2011).

**Box 1.**

**Computational Models of Social Interdependence and Preferences**

Van Winden and colleagues provided a computational model of the formation of social ties among interacting strangers (Bault et al., 2017; Sonnemans et al., 2006; Van Winden et al., 2008). In reduced form, it models social-tie formation between an individual *i* and another individual *j* with a dynamic weight γ_it_ attached to *j*’s utility in *i*’s own utility function for payoff *x: U*(*x*_i_) = (*s*_i_ − *c*_i_) + *b*_j_. Parameter *s*_i_ denotes the value from private resources, *c*_i_ is the cost of helping individual *j*, and *b*_j_ is the value derived from the donation made by the interaction partner. Thus, in our imaginary example of two strict payoff-maximizing individuals who do not help, *s*_i_ > 0, *c*_i_ = 0, and *b*_j_ = 0. However, when *c* ≤ *b*, the individual who benefits from being helped is (*s*_i_ − *c*_i_ + *b*_j_) ≥ *s*_i_.

Individual *j*’s helping provides “impulse” *I* on the individual *i*’s utility, which is negative when the benefits from others’ helping fall short of the costs of helping the other (i.e., when *c*_i_ > *b*_j_) yet positive when the benefits from others’ helping exceed the costs of helping the other (i.e., when *c*_i_ < *b*_j_). *I* conditions weight γ_it_ on any subsequent interaction *t* + 1: *γ*_it + 1_ = ƒ(*γ*_it_, *I*) (with −1 ≤ *I* ≤ 1). For any subsequent interaction, the individual’s utility is then assumed to incorporate γ: *U*_it + 1_ = *s*_i_ − (*c*_i_ − *γ*_it + 1_) + *b*_j_. In this formulation, the cost of helping individual *j* is reduced when there is a positive tie resulting from individual *j* helping on the previous round of interaction, and helping individual *j* becomes less costly and thus more likely.

Social-tie formation can shape social preferences that, in turn, can be formally modeled in a utility function in which the individual’s utility from decision-making not only depends on the (expected) value to oneself but also on the (expected) value to interdependent others. One widely used computational model is the one developed by Charness and Rabin (2002): *U*_i_ (*x*_i_, *x*_j_) = (1 − *w*)*x*_i_ +*wx*_j_, with *w* = α(1 + γ*a*)ρ *+* β(1 *+ γb*)σ, where *x*_i_, *x*_j_ is individual’s *i* and *j*’s payoffs and *w* is the weight that *i* puts on *j*’s payoff (with 0 ≤ *w* ≤ 1). Full selfishness of *i* would imply *w* = 0 (as in our imaginary example of payoff-maximizing strangers).

The indicators α and β are used to discern between *i* being ahead of *j* in terms of payoff (i.e., α = 1 if *x*_i_ > *x*_j_ and α = 0 otherwise) and *i* being behind *j* (i.e., β = 1 and *x*_i_ *< x*_j_ otherwise). The parameters ρ and σ then measure altruism and spite, respectively, with ρ > 0 indicating individual *i*’s positive concern for individual *j* and σ > 0 indicating that individual *i* dislikes being behind individual *j* (i.e., inequity aversion; Fehr & Schmidt, 1999; Loewenstein et al., 1989).

Indicator γ (with −1 ≤ γ ≤ 1) in the specification of *w* can be used to capture the impact of social ties between *i* and *j*. Accordingly, *a* and *b* capture the change in altruism and spite, respectively, when *i* faces a partner with more positive and negative social ties.

Second, and relatedly, cooperation can be reinforced via indirect reciprocity and reputation-based partner’s choice (Nowak & Sigmund, 2005; Roberts et al., 2021). Reputation can spread via gossip or observability and be sustained by social norms (e.g., stern judging; see Santos et al., 2018) and properties of social networks that afford ostracizing and selecting cooperative partners (Gallo & Yan, 2015; Giardini & Vilone, 2016; Okada, 2020; Takacs et al., 2021). Through gossip, for example, people can exchange information on the trustworthiness and cooperativeness of interaction partners. Behaving cooperatively gives individuals a reputation of being a cooperator rather than a defector that is then transmitted in a social network (Hammond & Axelrod, 2006b; Mifune et al., 2010; Romano, Giardini et al., 2021). Accurate transmission of reputation information is key for sustaining indirect reciprocity and reputation, because people might also be tempted to provide dishonest information for personal interest (Wu et al., 2021). When reputation is at stake and transmitted accurately enough, behaving cooperatively is advantageous: Others are more likely to seek partners with a cooperative reputation and are more willing to cooperate themselves to avoid abandonment (Barclay, 2016; Gross & De Dreu, 2019a; Stallen et al., 2023; Nowak & Sigmund, 2005; Romano et al., 2022; Fig. 3b).

### From Coalitions to Stable Groups: Social Preferences and Group Identification

The downstream consequences of helping and reciprocity can make cooperation the likely default. Across repeated interactions and generations, cooperation becomes a beneficial strategy and over time instills socially shared norms that may be applied also with new partners one has no knowledge about. Such generalized expectations can already emerge after a few interactions (Rojek-Giffin et al., 2023) but can also be part of a complex socialization process that is transmitted and shaped over generations (Farina et al., 2021; Hare, 2017; Rand & Nowak, 2013). Either way, through generalized norms, individuals develop social preferences for cooperation that, to some degree, become independent of others’ reputation or group membership (see also Box 1) and how one’s own and others’ payoffs are correlated. Indeed, experiments confirm that individuals with prosocial preferences more likely cooperate in one-shot hawk-dove games than those with selfish preferences (e.g., McClintock & Liebrand, 1988; Thielmann et al., 2020).^1^

At its core, groups are “containers of bounded generalized reciprocity” (Yamagishi & Kiyonari, 2000). Built on positively interdependent individuals who learned the value of reciprocal cooperation in repeated interactions (Aaldering et al., 2018; Balliet et al., 2014; Bicchieri, 2005; Yamagishi & Mifune, 2009; Fig. 3c). Understanding group formation and the evolution of cooperation in terms of fitness interdependency and reciprocity require (rudimentary) cognitive abilities such as recognition, memory, and language (Crowley et al., 1996; Tomasello et al., 2005) but not complex assumptions about social perceptions and motivations often seen in social-psychological theory on groups and collectives (e.g., Tajfel & Turner, 1979; Turner, 1985). In fact, our ultimate analysis can inform our understanding of social perceptions, preferences, and expectations within existing groups. For example, a reciprocity and norm-based account can explain why cultural variation often is (perceived to be) smaller within rather than between groups (Antal et al., 2009; Axelrod et al., 2004; Hammond & Axelrod, 2006b; Krupp et al., 2008; Mathew & Perreault, 2015; Yzerbyt et al., 2001). Over time, (in)direct reciprocity, reputation, and (enforcement of) group norms can give rise to cultural practices and communication patterns—including nonverbal signals, group tags, and spoken language—that are group-specific and may not be easily understood by outsiders. Implicit rules of engagement—what is appropriate or unacceptable and how and what to communicate—evolve in the context of and are shaped by direct and indirect reciprocity, ultimately leading to socially shared norms akin to a “secret code that is written nowhere, known by none, and understood by all” (Sapir, 1927, p. 32).

That cultural homogeneity is a consequence rather than cause of reciprocity, and reputation-based partner selection provides an alternative to the idea that “birds of feather flock together” because of homophily preferences (Melamed et al., 2020; Romano et al., 2017; Traulsen & Claussen, 2004), self-categorization (Turner, 1985), and attraction-selection-attrition dynamics (Schneider et al., 2013). These perspectives suggest that cultural homogeneity emerges because humans preferentially select themselves into groups of similar rather than dissimilar others, because groups more likely recruit new members that are more rather than less like the “prototypical” group member, and because individuals who are or develop more similar to the average group member are less likely to leave the collective (i.e., person-organization fit; Kristof, 1996). Although descriptively valid, these perspectives do not explain how groups form ex nihilo or why similarity attracts. Viewed from the perspective of fitness interdependence and reciprocity, local group cultures in which individuals are comparatively similar in both thinking and doing are the endogenous result of reciprocal cooperation and reputation-based partner choice (Gallo & Yan, 2015).

Related to work on homophily and selection-attraction-attrition is the literature on the psychological states associated with group membership such as group commitment (Dutton et al., 1994), perceived cohesiveness (McConnell et al., 1997; Grossman et al., 2022), and social identification (Hogg & Terry, 2000; Spears, 2021). Therein it often is assumed—implicitly or explicitly—that these group psychologies are pivotal drivers of how helpful individuals are (Penner et al., 2005), how much effort they exert toward group goals (Ellemers et al., 2004), and how aligned affective and neural processes between group members are (Hu et al., 2017; Yang et al. 2020). As with homophily and attraction-selection-attrition perspectives, however, this literature cannot explain how strangers form groups and thus what individuals can identify with and commit to. In fact, it cannot be excluded that (self-reported) identification and perceived cohesion are consequences rather than causes of strangers initiating and reciprocating cooperation (De Dreu, Farina, et al., 2022; Dunham, 2018; Grimalda et al., 2018). If true, (self-reported) group psychologies may often be an epiphenomenon or consequence rather than cause of reciprocal cooperation and reputation-based partner selection.

## Evolving Complex Social Structures

Small coalitions can grow into larger groups by selectively integrating outsiders with a reputation for being cooperative (Bergmuller et al., 2007; Gallo & Yan, 2015; Gross & De Dreu, 2019a; Kokko et al., 2001; Schneider et al., 2013) and by merging with other groups. Either way, helping within larger groups and collectives can be directly aimed at other individuals or take a more general form of making costly contributions to common goals, thereby creating group-level club goods—goods that are shared across group members but require costly contributions to maintain them, such as collective safety, alloparenting, or food sharing (Smaldino, 2014, 2019). Club goods allow individuals to use and combine individual resources—insights, skills, efforts—for the greater benefit of the entire group (Gross & De Dreu, 2019b). For example, rather than standing on guard so that one’s family can sleep, individuals can collectively build a wall that prevents predators from entering the village (Gross & De Dreu, 2019b; Gross et al., 2020; Mayshar et al., 2022).^2^

Club goods enable cooperation to transcend dyadic interactions and create benefits for all group members. Club goods also create collective-action problems that groups need to manage. First, some individuals may benefit less from (some) club goods than others and may thus be less motivated to contribute (Gross & Böhm, 2020; Hoenig et al., 2023a; Van Dijk & Wilke, 1993). Second, not only contributors but also noncontributors benefit from club goods—even those who did not contribute to building a wall are protected against dangerous predators. Group members may thus be tempted not to contribute for personal gain or because they fear others may not contribute (i.e., free riding). The problem that is encountered in dyadic interactions is also present at the group level. Third, some club goods already yield benefits when some but not all members contribute (e.g., step-level public goods and snow-drift games; Van Dijk & De Dreu, 2021). Once a wall around the village is high enough to keep predators out, adding further effort is wasteful. In such cases, groups not only need to deal with potential free riding but also with problems of coordination—who contributes what, where, and when (Gross & De Dreu, 2019b; Schelling, 1960; Van Dijk & De Dreu, 2021).

Regardless of the structural features of club goods, establishing them requires groups to define who is part of the group and can benefit from club goods and who is not. It can lead to sharply defined and enforced group boundaries, alongside a demand to identify and separate in-group from out-group members (Brewer, 1999). With club goods being established and individually beneficial, staying close to others and thinking and behaving like others are basic means to ensure one is being identified as “in-group” and a potential beneficiary of the group’s club goods.

### Governing the Commons and the Emergence of Social Institutions

In smaller groups, individuals can monitor and track whether and how much others contributed to the group’s club goods. In such cases, individuals use a range of informal measures to solve problems of cooperation via reciprocity and reputation. They can express anger at free riders (De Melo et al., 2014; Van Kleef et al., 2010), punish or ostracize norm violators (Fehr & Gächter, 2000; Mathew & Boyd, 2011; Stallen et al., 2018), gossip about an individual’s dishonest character (Feinberg et al., 2014; Molho et al., 2020; Sommerfeld et al., 2007), and reward others for being cooperative and loyal (Molenmaker et al., 2014).

Emotional expressions, punishment, and gossip all signal that cooperation is valued and that defection and free riding are not. These social signals socialize and sometimes coerce individuals into prosocial and norm-abiding group members (Molho et al., 2020) and make free riding more costly and cooperation comparatively more attractive from a strict individual payoff-maximizing perspective (Fehr & Gächter, 2000).^3^ Indeed, groups better maintain their club goods when group members can gossip about and punish free riders (Balliet et al., 2011), express anger at wrongdoers (Pietroni et al., 2008; Pillutla & Murnighan, 1996), and provide symbolic or material rewards to cooperators (Molenmaker et al., 2014).

**Fig. 4.**
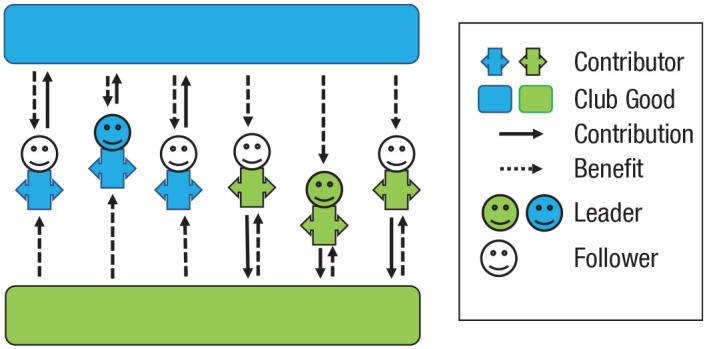
Multilayered collective with multiple club goods (blue and green rectangles), role specialization in which some group members are “responsible” for blue club goods and other group members are responsible for green club goods, and vertical specialization in leader (solid faces) and follower structures.

In larger groups, social monitoring can become difficult, and expressing anger, gossip, or reward cooperation may not be adequately and accurately applied at scale. As groups increase in size and develop and maintain multiple club goods (Fig. 4), informal measures can lose their effectiveness and become increasingly replaced by “institutionalized” measures to deter free riding and facilitate collective action (Bicchieri, 2005; Powers et al., 2016). Examples of such “institutions” include the codification of rules (e.g., rule of law), centralized punishment systems (Baldassarri & Grossman, 2011; Gross et al., 2018; Yamagishi, 1986), appointment of leaders (Van Dijk, Wilke, & Wit, 2003), contractual assignments of individuals to roles and tasks (Simon, 1947), and guided socialization of prosocial values and norms through spiritual teaching and formalized education systems (Eisenberg et al., 2014). These and related institutions can thus all be considered adaptive responses to problems of cooperation and coordination inherent to increasingly complex social organization. There is indeed good evidence that collective action in larger groups is coordinated more efficiently and to collective benefit in groups with rather than without vertical (leader-follower) and horizontal (role and task) specializations (De Dreu & Triki, 2022; Gavrilets & Fortunato, 2014; Smith et al., 2022).

Social institutions often become club goods in themselves (Yamagishi, 1986). Individuals need to contribute energy to support, administer, and execute their social institutions (e.g., by paying income tax, casting votes in elections, or volunteering initiatives). In addition, individuals need to comply with institutional directives and follow rules that may go against their immediate self-interest. This explains why individuals do not always comply with rules such as waiting for a red light, paying taxes, or telling the truth (Gross & Vostroknutov, 2022; Shalvi et al., 2011). It also explains why individuals sometimes punish lying and deception but also, when doing so is in their immediate self-interest, tolerate or even collude with others’ deceitful initiatives (Gross et al., 2018; Gross & De Dreu, 2021; Weisel & Shalvi, 2015). Accordingly, groups often organize additional systems for monitoring and control—collectives not only design laws but also law enforcement, and internal-affairs units that control law-enforcement agencies, and so on, creating multilayered institutions of increasing complexity.

Multilayered collectives with multiple club goods and horizontal and vertical role specializations are inherently fragile. Role specializations create local subgroups within which interdependencies are likely to be stronger and reciprocal interactions more frequent than between subgroups (Fig. 4). Both leaders and their followers may benefit those within their subgroup more readily than those outside their subgroup, and such parochialism can undermine cohesion and cooperation with the overarching collective (e.g., Dovidio et al., 1998; Gross et al., 2023; Molenmaker et al., 2023). Likewise, some club goods within multilayered collectives may benefit some group members more than others, and cooperation can thus create wealth disparities within the overarching collective (Gross et al., 2020; Hoenig et al., 2023a, 2023b). Feelings of deprivation and envy within disadvantaged subgroups can set the stage for protest and conflict, ultimately leading to the disintegration of the overarching collective.

## Fission–Fusion Dynamics in Multilayered Collectives

Conceptualizing human social organization as a bottom-up process grounded in fitness interdependence, reciprocity, and reputation-based partner selection allows group formation and the emergence of comparatively homogeneous group cultures to be understood independent of the presence or absence of outside groups. Still, outside groups exist because they developed independently, or because original “parent” groups split into different groups. An example of the latter is the Bandkeramik culture (5600–4900 BC). Originating in what is now the Czech Republic, over the course of generations it spread across central and western Europe. Archaeologists converge on the idea that villages growing to a certain size tend to split, with some villagers moving into new territory to practice agriculture until a certain size was reached, on which new groups would split off again and move into yet to be cultured lands, and so on (Bentley et al., 2012; Gronenborg et al., 2014; Meyer et al., 2018).

For most of its existence, parent and offspring communities of Bandkeramik peacefully coexisted with presumably infrequent intergroup contact likely geared toward mutually beneficial trade. However, and possibly because population growth alongside harshening climate reduced food supply, peaceful coexistence increasingly gave way to intergroup conflicts and coordinated violence (Meyer et al., 2018). As such, the Bandkeramik is a prime example of intergroup relations oscillating between peaceful coexistence and mutually beneficial trade, and polarization and intergroup conflict. It also illustrates how external factors that create resource scarcity and carrying-capacity stress—situations in which resource supplies fall short of what groups need to function—alter fitness interdependencies not only within but also between groups (De Dreu et al., 2020; De Dreu, Gross et al., 2022; Read & LeBlanc, 2003).

### Intergroup Interdependence and Parochialism

As for interactions between single individuals, intergroup relations are shaped by social interdependencies and the “game” groups of individuals are engaged in. Negative interdependencies between groups resulting from, for example, resource scarcities and deteriorating habitats, likely drive groups toward competition and attempts to exploit (individuals in) neighboring groups (Brown et al., 2022; De Dreu et al., 2020; De Dreu, Gross et al., 2022; Rodrigues et al., 2022). Conversely, shared ancestry, a common enemy, or the presence of mutually beneficial trade opportunities create positive intergroup interdependencies and drive groups toward reciprocal cooperation. In the extreme, this can lead to the merging of groups of individuals into larger, multilayered collectives such as hamlets and villages merging into cities and small firms merging into multinational companies.

What often goes unnoticed is that for intergroup interdependencies to shape intergroup relations, there need to be repeated opportunities for interactions across group boundaries (Gross et al., 2023; Paluck et al., 2021). When intergroup interactions are absent or unlikely, cooperation is bound to be parochial and limited to other members of one’s own in-group (Table 1). In such situations, intergroup relations most likely are marked by a “live-and-let-live” coexistence. Intergroup interdependencies become critical when cross-boundary interactions are increasingly frequent and opportunities for intergroup cooperation, reciprocity, and reputation-based partner choice increase. Specifically, when intergroup interdependencies are positively skewed (i.e., payoffs to individuals from different groups are positively correlated), parochial cooperation may increasingly give way to universal cooperation in which individuals do not discriminate in costly helping and reciprocity between in- and out-group members, and group-specific club goods merge into public goods that serve in- and out-group members alike (Gross et al., 2023; see also Binder et al., 2009; Pisor & Surbeck, 2019). Over time, group boundaries fade, and groups may fuse into larger collectives. Higher relational mobility and shared environmental pressures such as climate change may thus foster group fusion.

**Table 1.** Intergroup Behavior and Outcomes as a Function of Intergroup Interdependence and Interaction Frequency

Intergroup interaction	Intergroup interdependence		
	Negative sum	Mixed motive	Positive sum
Limited			
Behavior	Parochial cooperation	Parochial cooperation	Parochial cooperation
Outcome	Coexistence	Coexistence	Coexistence
Frequent			
Behavior	Competition	Parochial cooperation	Universal cooperation
Outcome	Polarization/conflict	Transactional exchange	Integration/fusion

Note: Intergroup interaction is defined as the likelihood that in-group individuals can engage in costly helping of out-group individuals (e.g., see Gross et al., 2023); parochial cooperation (competition) is defined as the tendency to preferentially cooperate (compete) with in-group (out-group) individuals; universal cooperation is defined as the tendency to cooperate with both in- and out-group individuals; and transactional exchange is defined as the explicit negotiation between individuals about what each give and take.

When, in contrast, intergroup interdependencies are negatively skewed and payoffs to individuals from different groups are negatively correlated, we would not only expect parochial cooperation within groups but also competition between groups (De Dreu, Gross et al., 2022). Indeed, in the case of negative intergroup interdependencies, hurting outsiders undermines the out-group’s capacity to work together and indirectly increases the in-group’s chances of prevailing. Over time and repeated interactions, such intergroup competition and conflict can also bind individuals within groups and reinforce parochial cooperation (e.g., Choi & Bowles, 2007)—intergroup conflict not only emerges from but also shapes the interdependence structure within and between groups.

Experiments support the conjecture that intergroup interactions depend on intergroup interdependencies. For example, Bornstein and Gilula (2003) examined intergroup competition in intergroup hawk-dove and stag-hunt games, in which payoffs to in- and out-group members are more negatively versus more positively correlated. Results showed more competition and conflict in intergroup hawk-dove than stag-hunt games. More generally, experiments typically observe more out-group aggression when payoffs to in- and out-group members are negatively rather than positively correlated as in, for example, attacker-defender contests on the one hand and nested social dilemmas on the other (De Dreu et al., 2020; Weisel & Zultan, 2021).

There is good evidence that, all else being equal, lone individuals more likely cooperate than individuals nested in groups (McCallum et al., 1985; Wildschut et al., 2003). This *interindividual–intergroup discontinuity effect* implies that even when payoffs to in- and out-group individuals are positively correlated, and cross-boundary interactions are frequent, groups merging into overarching collectives may be less likely than “lone” individuals forming coalitions and groups. There may be two reasons for this. First, individuals interacting across group boundaries are not only interdependent with their out-group partner but also with other members of their respective in-groups. This can mean that assets spent on helping outsiders cannot be used to benefit (members of) the “in-group”—to at least some extent, helping outsiders impoverishes the in-group (Brewer, 1999). Second, and relatedly, social institutions for governing club goods are typically group-bound; peer punishment, leadership, the rule of law, and norm socialization are all concerned with the maintenance of local club goods and reduce the risk of being exploited by noncooperative insiders. Institutions typically do not reach outsiders as effectively and are less able to reduce the risk of being exploited by outsiders as much as they reduce the risk of being exploited by insiders. Accordingly, individuals nested in groups should be less inclined to help outsiders than would lone strangers (i.e., parochial cooperation; Balliet et al., 2014; De Dreu, Farina et al., 2022; Lane, 2016; Romano, Sutter, et al., 2021). Indeed, individuals trust outsiders less than insiders, expect that outsiders may exploit rather than reciprocate cooperation, and are more willing themselves to exploit outsiders rather than insiders (Balliet et al., 2014; Romano et al., 2017). Alone and in combination, such parochialism explains the interindividual–intergroup discontinuity effect, how insiders become seen more favorably than outsiders, how individuals gravitate towards us-versus-them thinking (Yamagishi & Mifune, 2016), and why group fusion may be more difficult than group fission.

## Conclusions

With increased mobility and globalization, individuals more likely meet and interact with strangers they have never met before and may never meet again (Buchan et al., 2009). And yet, depending on the perceived correlations between their individual payoffs—their perceived fitness interdependence—strangers may help and return the favor, seek each other out again on future occasions, and form stable coalitions and groups marked by reciprocal cooperation instilled in cooperation norms, other-regarding preferences, and shared notions of fairness. Over time, groups as containers of bounded generalized reciprocity develop local cultures with social institutions to govern the commons—increasingly complex social structures are built atop informal systems of (in)direct reciprocity that become more difficult to maintain as groups increase in size and complexity. At the same time, human social organization is inherently parochial. Although this benefits individuals and their groups, it impedes the merging of groups into overarching collectives and potentially leads to conflict. Intergroup fusion requires intergroup interdependencies that are positively skewed, frequent cross-boundary interactions, and institutionalized measures against free riding that reach across group boundaries.

The current analysis invoked theories that refer to long timeframes that include multiple generations of individuals who shape and contribute to existing groups in which they are born or recruited (for related analyses in other species, e.g., see Kappeler & van Schaik, 2002; Rodrigues et al., 2022; Smith et al., 2022). Evolutionary theory proposes that mechanisms that increase individual fitness may be favored over those that do not, or even reduce individual fitness. Accordingly, over generations of group formation and group living there likely is cultural and biological selection for propensities for altruistic helping, (in)direct reciprocity, and creating and adhering to social institutions for governing the commons. At the same time, our framework can also inform how groups forms, develop, and change over shorter periods of time. For example, the threat of global warming drastically changes perceived interdependencies among individuals, and this favors the formation and institutionalization of new collectives (e.g., Friday for Future).

Reciprocity and reputation-based partner selection explain “bottom-up” social organization, including the emergence of role divisions and hierarchical structures. Group leaders, unit managers, and governmental officials in turn impact social organization in a “top-down” fashion (Gelfand et al., 2012; King et al., 2009; Lehmann et al., 2022). As such, our framework reveals how bottom-up social organization is intimately entwined with and feeds top-down organization spearheaded by “group leaders.” Finally, our framework reveals how social interactions within and between groups gives rise to a range of well-documented psychological states, from social preferences and group identification to emotion signals and us-versus-them thinking. Although often studied in isolation, our framework reveals these group psychologies share a common function in the management of collective-action problems inherent to group living. Because indeed, when groups solve their collective action problems well, their individuals benefit from sustained club goods and the joy of being surrounded by cooperative others.

## Transparency

*Action Editor:* Henrik Olsson

*Editor:* Interim Editorial Panel

*Author Contributions*

C. K. W. De Dreu, J. Gross, and A. Romano conceived the project and wrote the manuscript. All of the authors approved the final manuscript for submission.

## Notes

1. Stable interindividual differences in social preferences suggest that cooperation norms cannot fully replace reciprocity mechanisms and are not always fully internalized by all group members. Furthermore, cooperation norms need to combat the selfish incentives to free ride, and for various reasons some individuals may be predisposed to prioritize personal payoffs over collective interests and compliance with group-based norms for cooperation. Accordingly, reciprocity opportunities, reputation concerns, and partner choice may always be needed to “support” or enforce norms and likely do not become obsolete once groups establish shared social norms.

2. Club goods take the form of provision and maintenance club goods. Provision goods, also known as give-some dilemmas, require individuals to make costly contributions to club goods that do not exist ex nihilo, such as coordinating forces on a hunting party or building a wall around a village to keep predators out. Maintenance goods, also known as take-some dilemmas, allow individuals to privately consume from a common resource such as public health care and public parks. Provision goods pose the problem of not providing enough, and maintenance goods pose the problem of taking too much (Gaechter et al., 2022; Van Dijk & Wilke, 1993). Some, but not all, maintenance goods are the outcome of successful provision goods. An interesting question that we consider beyond the current scope is how humans create provision goods.

3. From this social-functions perspective on emotions, feelings such as regret, guilt, and shame are internal signals that one has violated cooperation norms that may endanger one’s reputation as a loyal and committed group member (Van Kleef & Lelieveld, 2022). There is indeed evidence that signaling regret and guilt serves an appeasement function and leads others to expect more forthcoming, cooperative behavior on future occasions (Van Kleef et al., 2010), reducing the likelihood of being punished for “bad” behavior and prompting more cooperation (Nunney et al., 2022).
